# Supplementary figures and images for: Cortical state contributions to neuronal response variability in the early visual cortex: A system identification approach
Source: PLoS Comput Biol. 2025 Nov 6;21(11):e1013661. doi: 10.1371/journal.pcbi.1013661 (PMC12614811; doi:10.1371/journal.pcbi.1013661)

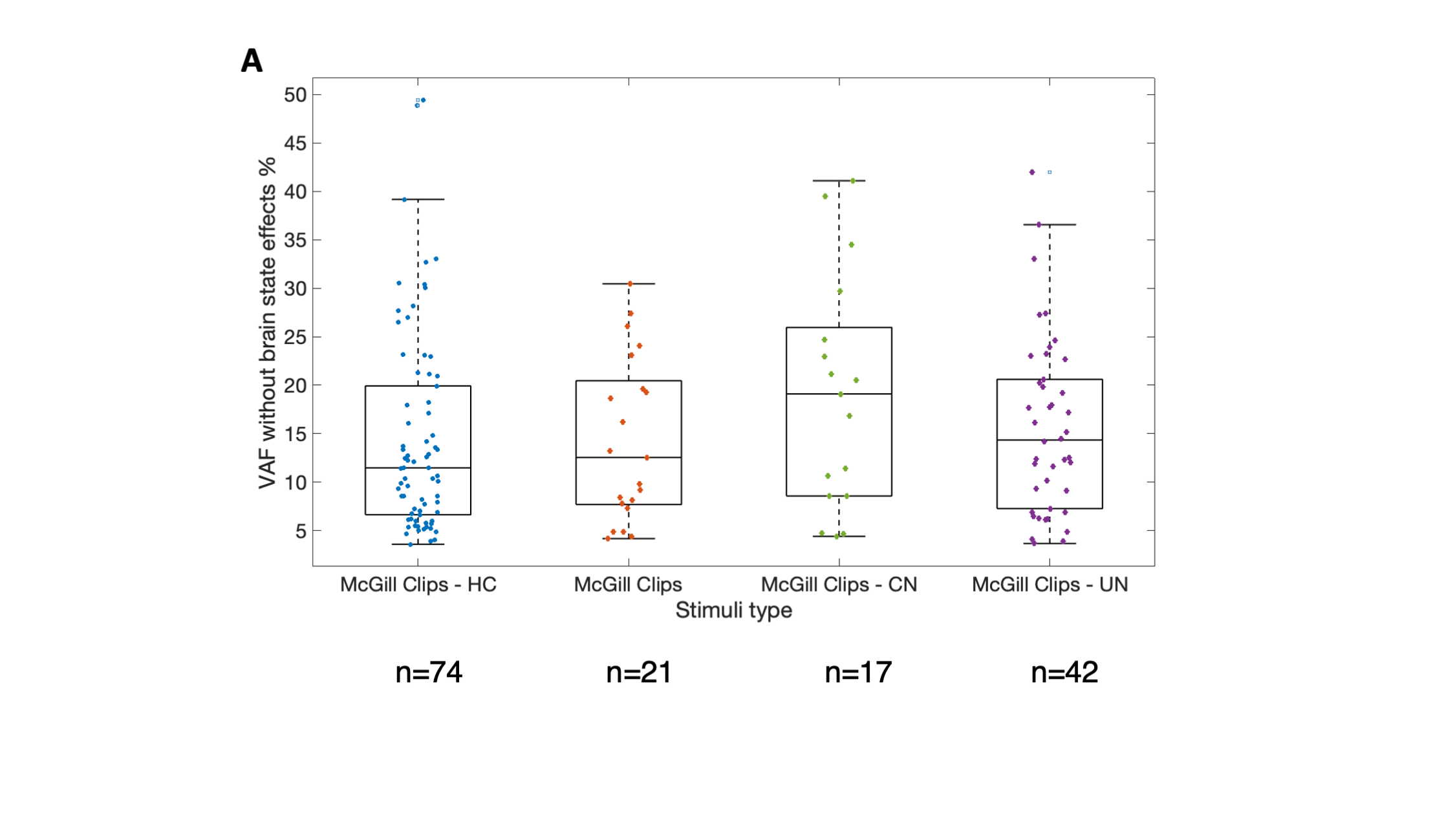

Supplement: S1 Fig — Each data point represents a neuron’s VAF accuracy obtained from system identification model with the stimulus-driven pathway only for the four types of stimuli. (TIF) [file pcbi.1013661.s001.tif]

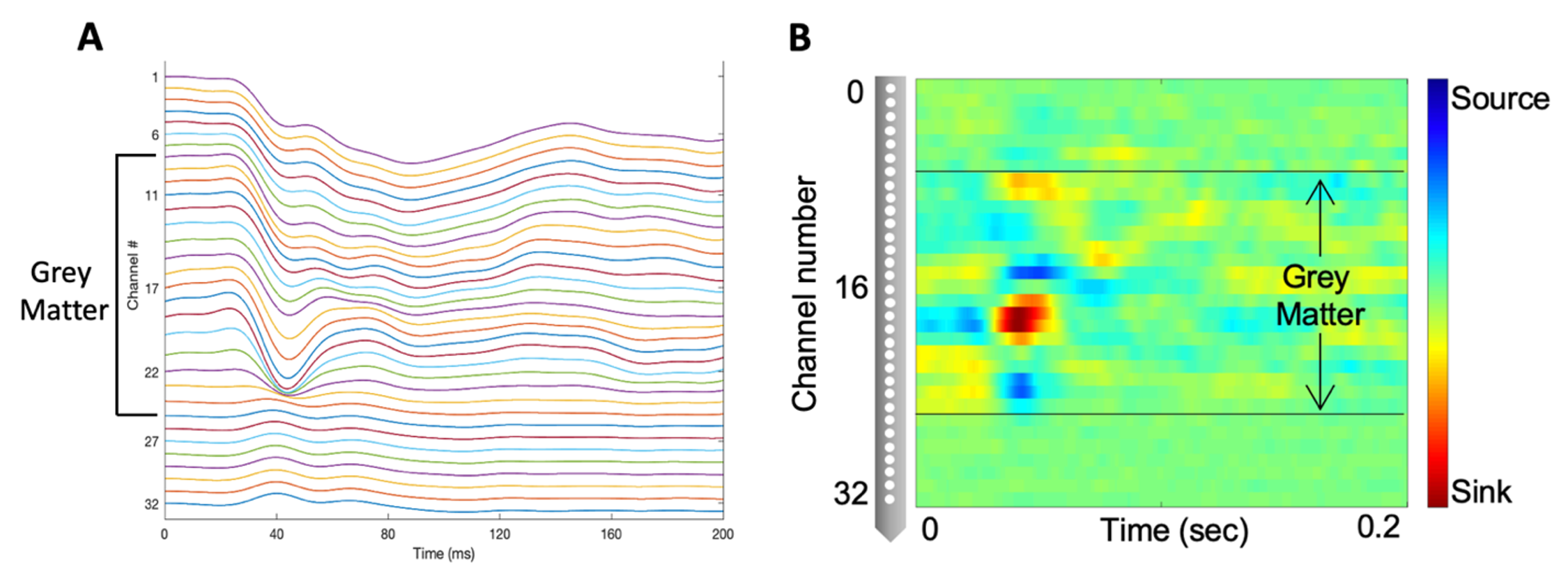

Supplement: S2 Fig — A) Raw LFP traces used to measure current source density (CSD) as a function of depth. We extracted LFP data (<100 Hz) from 32 channels while presenting sinewave gratings. We averaged the filtered LFP signals across trials to obtain the evoked response potential and used these to compute CSD. B) CSD plot generated using CSD plotter software [45]. Recording channels were 100 μm apart, so the apparent gray matter extended across about 1800 μm. Channels with a prominent current sink in the CSD profile were specified as the granular layer, and sources above and below this sink were identified as the supragranular and infragranular layers respectively. (TIF) [file pcbi.1013661.s002.tif]

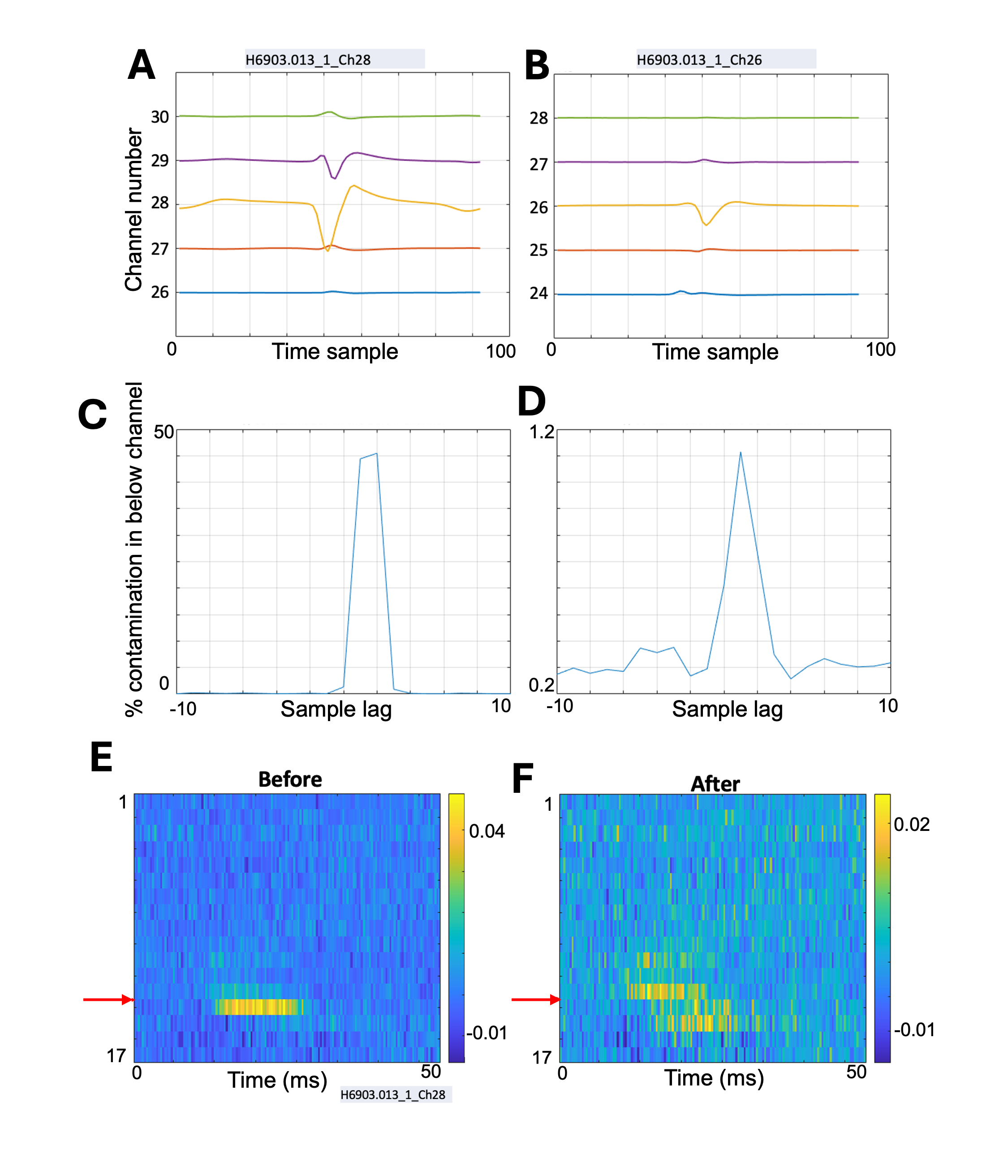

Supplement: S3 Fig — A, B) Spike waveforms recorded in adjacent channels for two example neurons. C and D) Percentage of spikes in the below channel contaminated with spikes of the neurons in A and B. To compute this, we count the number of spikes in the neighboring channel (i.e., channel located 100μm below, in this example) co-occurring with the neuron’s spikes from the primary channel (here, 28) from which it was recorded. If this count is > 10% of the total spikes in the neighbouring channel, we remove those spikes from the neighbouring channel. E) Estimated filter weights from MUA pathway when MUA from all the channels is used as inputs before removing spike contamination from the neighbouring channels. F) Same as E after removing spike contamination from the neighbouring channels. (TIF) [file pcbi.1013661.s003.tif]

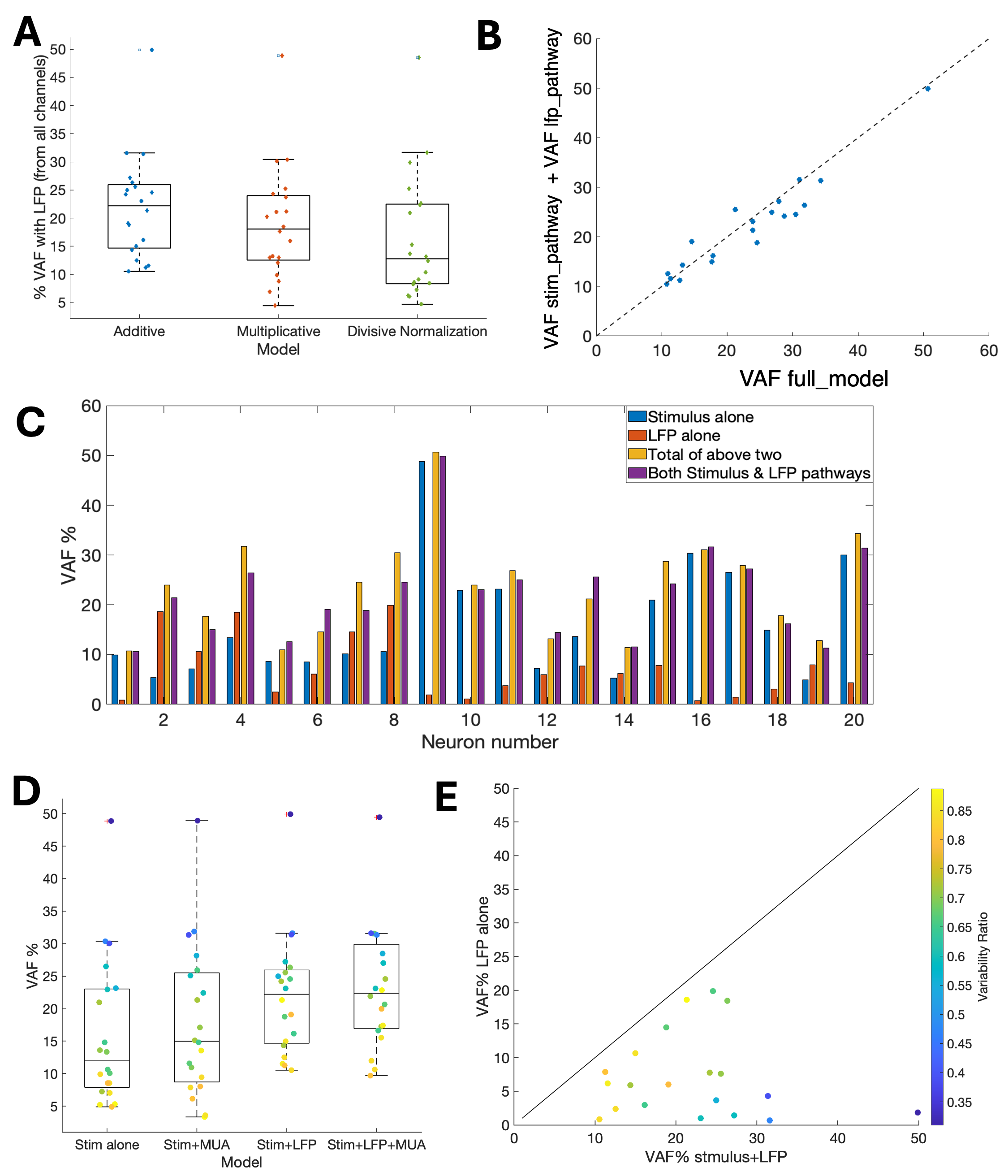

Supplement: S4 Fig — A) Box plot showing VAF from model with LFP from all channels acting additively: rest = N (rstim + mLFP), multiplicatively: rest = N (rstim * mLFP), or with divisive normalization: rest = N (rstim/ [semi saturation constant + gain*mLFP]). B) Scatter plot showing the summation of VAFs from stimulus pathway alone (VAF stim_pathway) and LFP pathway alone (VAF lfp_pathway) vs VAF from full model containing both stimulus and LFP pathways (VAF full_model). Dashed line indicates 1:1 relationship. C) Bar graph showing VAFs from stimulus pathway alone, LFP pathway alone, summation of both, and VAF from the full model containing both stimulus and LFP pathways. D) Box plot showing VAF from models; stimulus alone (mean VAF = 16.09%), Stimulus+MUA (mean VAF = 17.83%), Stimulus+LFP (Mean VAF = 21.97%) and Stimulus+LFP + MUA (mean VAF = 23.16%). Paired test: stimulus alone and stimulus+MUA = 9.0e-2, stimulus alone and stimulus+LFP = 5.5e-5. E) Scatter plot showing the VAFs from the model with LFP pathway alone and VAF from the model stimulus+LFP. (TIF) [file pcbi.1013661.s004.tif]

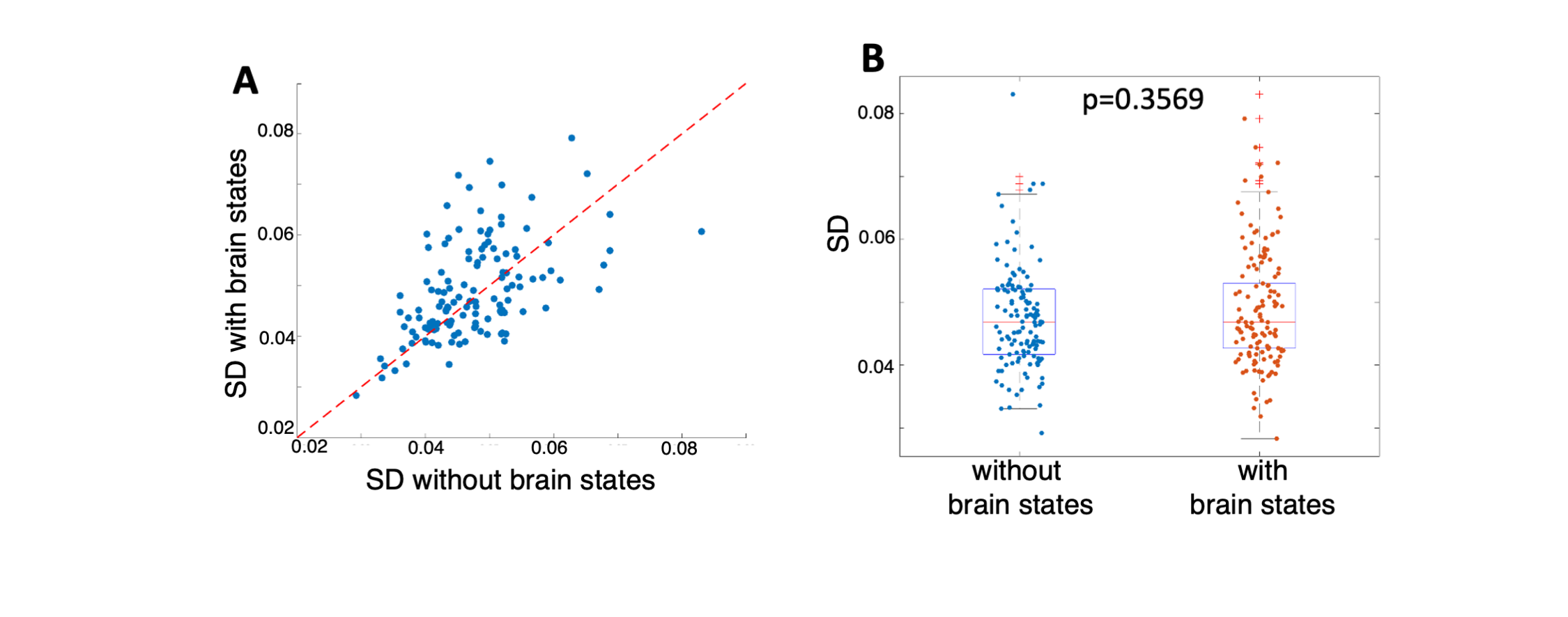

Supplement: S5 Fig — A) Scatter plot showing standard deviation of the zeroth time lag of the estimated receptive field with brain state pathways, vs. the same but for the base model without brain state. B) Distribution of standard deviations of the zeroth time lag of the estimated receptive fields without, and with, brain state models. (TIF) [file pcbi.1013661.s005.tif]

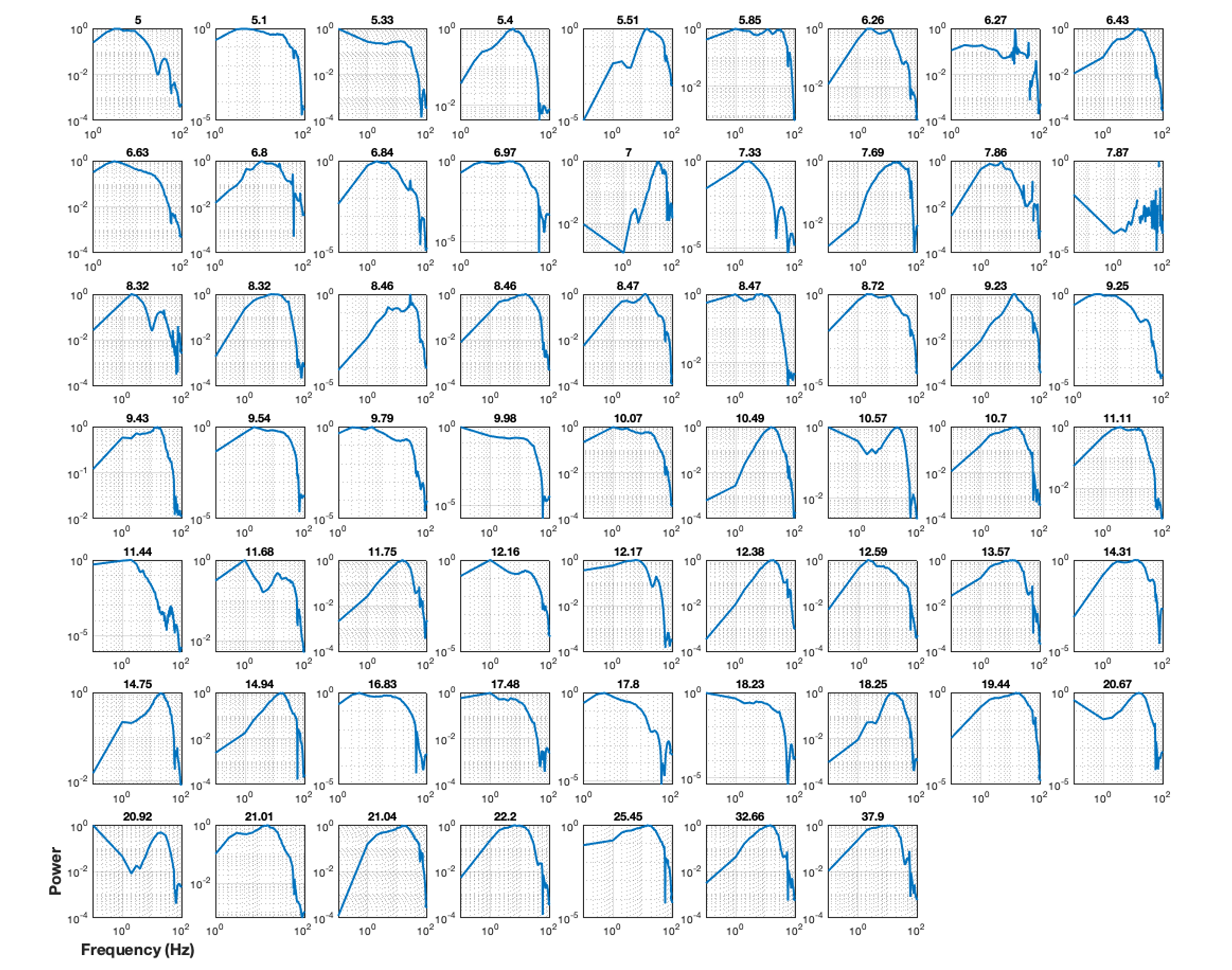

Supplement: S6 Fig — Power spectra of recorded LFPs filtered with estimated LFP temporal filters for neurons with >5% VAF improvements (n = 61). VAF improvements are reported on top of each power spectrum. (TIF) [file pcbi.1013661.s006.tif]

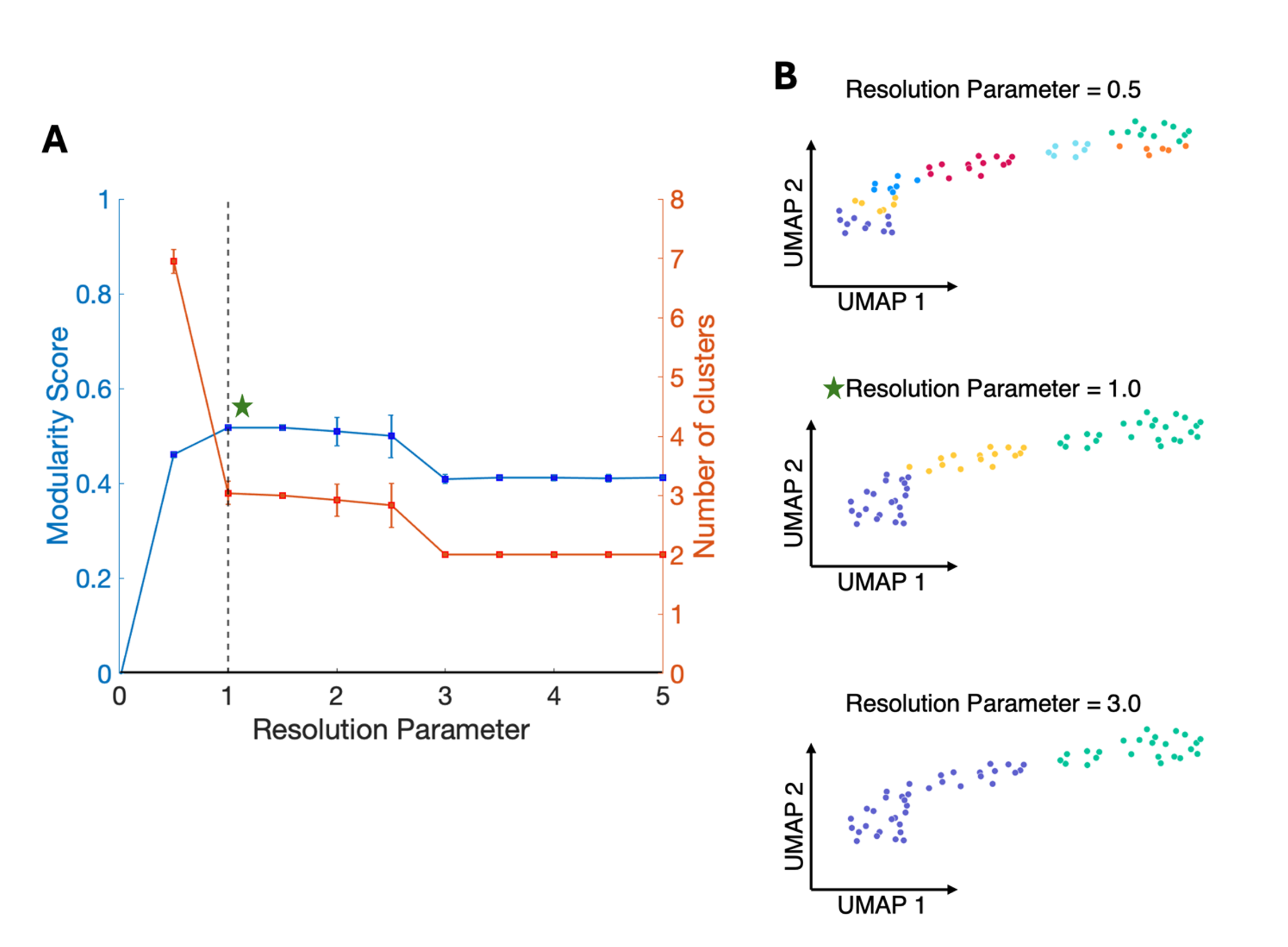

Supplement: S7 Fig — A) Modularity score and number of clusters vs resolution parameter in Louvain clustering. The dotted line when the modularity score is the highest at resolution parameter = 1.0 was selected for clustering. B) Clusters obtained with resolution parameter = 0.5, 1.0 and 3.0. Green star shows the clustering at the selected resolution parameter of 1.0. (TIF) [file pcbi.1013661.s007.tif]

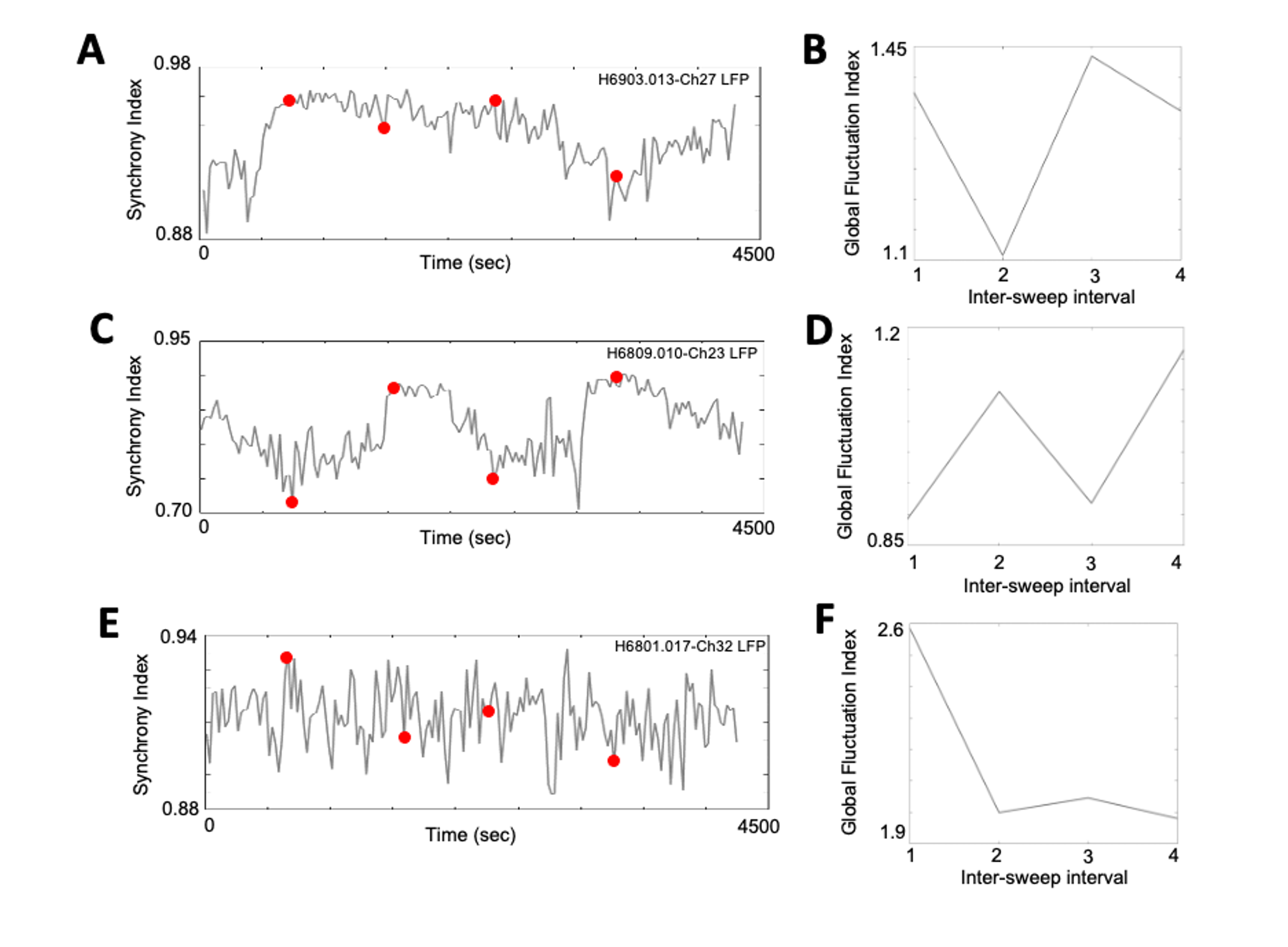

Supplement: S8 Fig — A) Synchrony index (SI) defined by equation 4 using PSD of a deep layer LFP signal. Red points indicate inter-sweep intervals. B) Global fluctuation index (GFI) defined by equation 3. C, E) Similar to A for another two datasets. D, F) Similar to B for another two datasets. The Pearson’s correlation between SI and GFI and its statistical significance for the three data recordings are r = 0.65, p = 0.35 (top row); r = 0.99, p = 5.9e-03 (middle row); and recording 3: r = 0.98, p = 1.7e-02 (bottom row). (TIF) [file pcbi.1013661.s008.tif]

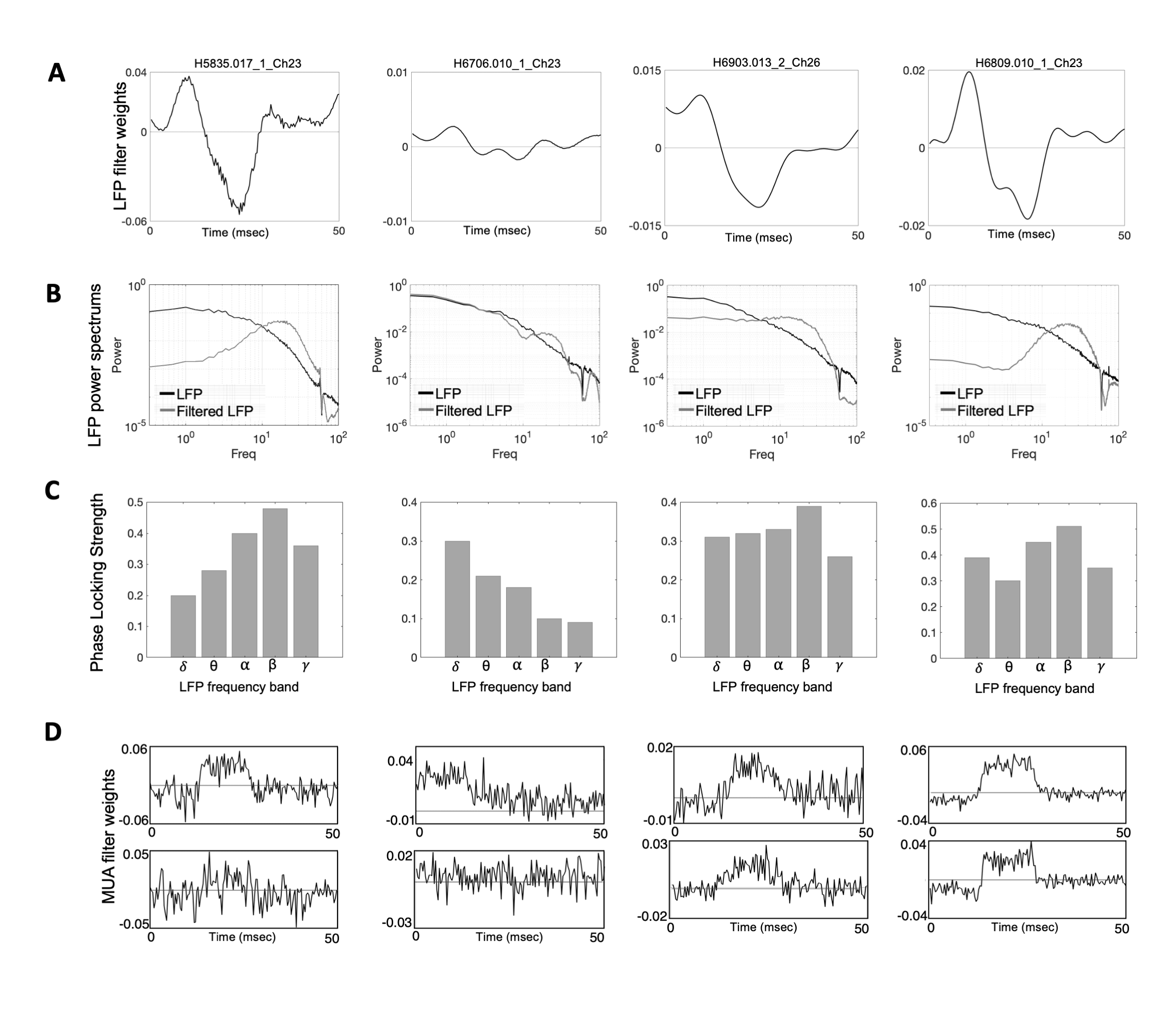

Supplement: S9 Fig — A) Estimated temporal filters from LFP-driven pathway for four example neurons when using LFP from the channel above. B) Input LFP (black) and filtered output LFP (grey) from the estimated temporal filters given in panel A. C) Phase locking strength (PLS) values (comparing spiking activity in relation to the phase of LFP bands). D) Estimated temporal filters from MUA-driven pathway for same example neurons when using MUA from the above and below channels. (TIF) [file pcbi.1013661.s009.tif]

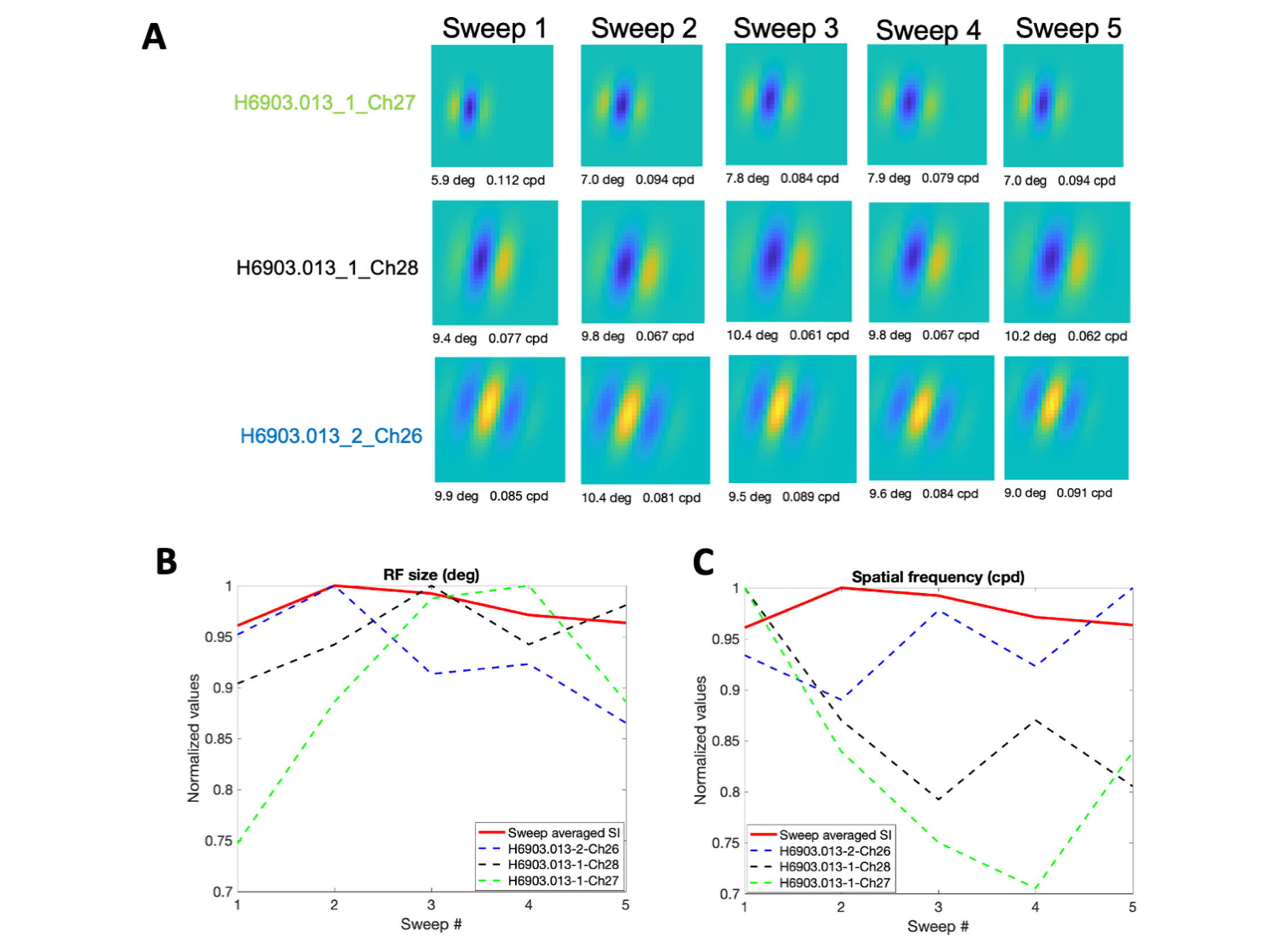

Supplement: S10 Fig — A) RF at the peak time lag of three neurons mapped across 5 sweeps. B) RF sizes 3 neurons in A across 5 sweeps in dashed lines and cortical state measure SI in red solid line. C) Similar to B, the optimal spatial frequencies of 3 neuronal RFs across 5 sweeps vs synchrony index (SI). RF sizes and optimal spatial frequencies were from best-fitting Gabor functions, as described in [34]. (TIF) [file pcbi.1013661.s010.tif]
